# Israeli mental health in the aftermath of the October 7 terrorist attack: risks, challenges, and recommendations

**DOI:** 10.1186/s13584-025-00682-8

**Published:** 2025-04-16

**Authors:** Yuval Neria, John C. Markowitz, Doron Amsalem, Yossi Levi-Belz, David Roe, Ido Lurie, Dana Tzur Bitan, Milton L. Wainberg, Shlomo Mendlovic

**Affiliations:** 1 New York State Psychiatric Institute, Department of Psychiatry, 1051 Riverside Drive, New York, NY 10032 USA; 2 Columbia University Vagelos College of Physicians & Surgeons, New York, NY USA; 3 The Lior Tsfaty Center for Suicide and Mental Pain Studies, Ruppin Academic Center, Emek Hefer, Israel; 4 Department of Community Mental Health, University of Haifa, Haifa, Israel; 5 Shalvata Mental Health Center, Hod Hasharon, Israel; 6 School of Medicine, Tel Aviv University, Tel Aviv, Israel

**Keywords:** October 7, 2023 terrorist attack, PTSD, Precision care

## Abstract

**Background:**

The October 7, 2023 terrorist attack and subsequent war in Israel have created an unprecedented mental health crisis. This commentary examines emerging data on the psychological impact of these events and argues for a paradigm shift in the Israeli mental healthcare system.

**Main body:**

Recent studies reveal a dramatic increase in PTSD, depression, and anxiety among the Israeli populace. These findings underscore the long-lasting and pervasive nature of psychological trauma. Certain populations are disproportionately affected: women, ethnic minorities (particularly Israeli Arabs, who comprise 18.1% of the population), and those experiencing traumatic loss, displacement, or economic hardship. These groups require prioritized and tailored interventions. While existing outcome research provides a solid foundation for treating common trauma-related disorders like PTSD, depression, and anxiety, the Israeli mental health system is ill-equipped to handle the surge in demand. Too many clinicians lack training in evidence-based trauma therapies and standardized assessments. To address this gap, we advocate a system-wide transformation. This involves widespread training in evidence-based assessments and time- limited therapies, a focus on precision psychiatry tailored to individual needs, and the implementation of task-shifting and task-sharing models to expand access to care.

**Conclusion:**

These strategies are crucial for mitigating the long-term mental health consequences of the October 7th attacks and fostering individual and societal resilience. Failure to act decisively will exacerbate the existing crisis, placing further strain on individuals, families, and Israeli society as a whole.

## Background

The October 7, 2023 attack was the worst terrorist event in Israeli history. Hamas invaders killed more than a thousand Israelis and took 251 into captivity [1, 2]. Subsequent lengthy military conflicts in the Gaza Strip, on the West Bank, at the northern border with Lebanon, and clashes with Iranian and Yemeni militants have caused further military and civilian casualties, injuring thousands. The conflict has displaced between 200,000 and 500,000 Israelis [3], producing instability and difficulties in returning home. These events have presented extreme psychosocial and economic challenges: losses, including homes and property; disrupted career trajectories and education; unemployment, fragmented social support, and disrupted communities.

Two recent articles in the Israel Journal of Health Policy Research. Katsoty and colleagues [4] utilized an evidence-based model predicting the prevalence of posttraumatic stress disorder (PTSD) following October 7. The model predicted that 5.3% (95% confidence interval, 1.64–9%, *N* = 519,923) of Israelis would develop PTSD from these adversities. In the second paper, Krivoy and Rosenthal [5] expressed doubt that the already undermanned, underfunded, and strained Israeli public health system is prepared to address the surge in mental health needs of the population facing the consequences of October 7 without undergoing systemic change.

We agree with Krivoy and Rosenthal that Israel needs to dramatically retool to face the oncoming tsunami of war-related psychiatric illness. In this commentary we discuss findings emerging from recent October 7 research, outline key challenges stemming from these.

findings, and call for a paradigm shift in the Israeli mental healthcare system in order to adequately respond to these challenges.

## Main body

### Vulnerable populations and disparities in impact

Large-scale terrorist attacks inflict severe mental health consequences on large populations [6]. The effects are particularly painful and disabling in highly exposed populations and often endure [7–9]. Previous research identified several key experiences that exacerbate the risk of mental health problems:

- *Immediate proximity* to traumatic events significantly increases the risk of PTSD, major depression, and anxiety disorders [10–12].
- *Traumatic and sudden loss of a loved one* can provoke prolonged sorrow, symptoms of major depression, and complicated grief [13, 14].
- *Forced displacement* is an acute stress that often induces anxiety and feelings of insecurity and instability, which may worsen or precipitate psychiatric problems [15].
- *Financial burdens* due to loss of job or income can evoke helplessness, exacerbating the risk of depression, and anxiety [16].
- *Moral injury* from experiencing betrayal by leaders and institutions [17] is associated with PTSD, depression and anxiety. A dramatic example is the extreme risk to mental and physical health of the hostages kidnapped on October 7. A slew of studies has documented broad, lingering, and disabling outcomes for hostages including psychopathology, functional impairment, and increased mortality [18–20].

Gender and ethnicity also influence mental health outcomes. A systematic review and meta-analysis found that women are more likely to develop PTSD and depression then men in war-afflicted regions [21]. Israeli women demonstrated higher vulnerability to traumatic stress-related symptoms following terrorist attacks on Israeli society in the early 2000s [22]. Similarly, ethnic minority groups face higher risk for adverse mental health outcomes after exposure to major traumatic events [23]. Several studies, including one conducted following the October 7 attack, show that Israeli Arabs, comprising 18.1% of Israel’s population (compared to 75% for Jews), carry increased risk for PTSD, depression, and anxiety [24]. Israeli Arabs have the same legal rights to mental health care as other citizens, but like many minoritized populations worldwide they experience bias, stigmatization, and discrimination that impede their access to and their quality of care [25]. State authorities often give lower priority to minority needs and ignore cultural competence [26], worsening coping strategies and psychological burden among Israeli Arabs compared to Israeli Jews [27, 28]. The post-October 7 Jewish-Arab conflicts further heightened this divide [29].

### The mental health impact

Studies conducted in Israel since the October 7 attacks have documented rising and persisting mental health problems, including PTSD, depression, anxiety [25, 30], suicide risk [31], substance use [32], and moral injury [17]. Findings from three prospective studies, conducted before and shortly after the attack and as recently as March 2024, provide the basis for our call to improve mental healthcare. The first study, by Levi-Belz and colleagues [33], used a longitudinal design in assessing a representative sample of 710 Israelis 6–7 weeks before and 5–6 weeks after October 7. They reported an almost doubling in rates of probable PTSD, from 16.2 to 29.8%. In the same three-month interval, prevalence of probable generalized anxiety disorder and depression increased from 24.9 to 42.7%, and from 31.3 to 44.8%, respectively. The findings underscored the need to provide rapid, nationwide assessments and triage for effective interventions to address these mental health needs of Israeli citizens.

Unfortunately, the response of the mental health community was partial at best, mostly providing treatment as usual, as most Israeli clinicians are not equipped to respond to extreme trauma [5].

Another longitudinal study [34] assessed PTSD, depression, generalized anxiety, functioning, and event centrality (the degree to which a traumatic event becomes a core part of an individual’s identity and life narrative), at approximately three (T1; *n* = 858) and seven (T2, *n* = 509) months post-attack, revealing high comorbidity among probable diagnoses of PTSD, depression, and anxiety; greater distress in women; and a dose-response relationship between severity of exposure and distress. Event centrality consistently predicted distress at both time points with contributions from gender and exposure. Whereas distress and event centrality remained stable, functioning improved significantly from T1 to T2, highlighting the different trajectories of distress and recovery.

Most recently, we conducted a three-wave study of 1,052 Israeli individuals residing in conflict zones beginning in February 2024, at five months (T1), six months (T2), and eight months (T3) after the attack. 75% of the sample reported above-threshold anxiety, depression, or PTSD symptoms at T1, 69% at T2, and 67% at T3. Individuals experiencing traumatic losses, forced displacement, or economic hardships consistently demonstrated higher rates across time points of probable anxiety, depression, and.

PTSD than those without such experience, and women more than men. Arab and other ethnic minorities reported higher anxiety and depression symptoms than Jews at every time point [18], consistent with previous October 7 research [25].

Thus research indicates that the October 7 attack and lengthy consequent war have had a major, ongoing psychopathological impact that is likely to persist. An already traumatized society faces new traumas. Aftereffects expectedly extend beyond PTSD to a range of psychiatric disorders and may well worsen suicidality [31] and substance use risk [32]. Gender and minority status, degree of exposure, and proximity to conflict zones matter: individuals with greater traumatic exposure, traumatic loss, forced displacement, or income loss face greater psychiatric risk. These factors indicate the profound mental health challenges individuals face in grappling with the effects of extreme war-related adversities.

### A call for systemic change

Effectively addressing these national problems requires taking proactive measures to provide the necessary effective care for individuals most at risk. Mental health providers should prioritize women and minority group members, those who have endured captivity, traumatic loss, displacement, and war-related economic and social insecurity, each of which raises the odds of symptoms and long-term chronic reactions. These stressors should serve as critical markers to help healthcare providers prioritize and tailor treatment efforts, ensuring that those in greatest need receive focused evidence-based and accessible care. Such targeted interventions are essential not only for the well-being of the affected individuals but also for broader societal health, the overall well-being and the resilience of families and communities.

Not all treatments are equal [33]. Psychotropic medications can improve mood and reduce anxiety symptoms but often do not help individuals address the changes in outlook and psychosocial disruptions trauma can bring [34]. Medication rarely brings about PTSD remission [35]. Nor are all psychotherapies equally effective for depression, anxiety, moral injury, substance use and PTSD [36].

Moreover, the Israeli mental health workforce is unprepared to address the crisis. Many if not most practitioners do not ask systematically about their patients’ experience of trauma, dissociation, and moral injury. They often do not focus on diagnosis, which should drive determination of treatment options (37–38). They ignore serial symptom assessment and feedback- informed approaches. Crucially, they lack training in the brief, structured, research-supported psychotherapies shown to effectively treat these disorders [39]. Applying the wrong, open-ended treatment can demoralize practitioners and patients, prolong suffering, increase costs, and severely limit access to mental health care.

These challenges demand an urgent effort to systematically transform trauma care in Israel. To effectively address sources of psychological strain, the Israeli mental health system should adopt care strategies including a structured diagnostic process, training in and prioritization of evidence-based, time-limited therapies, and clinical outcomes monitoring. Israel should ensure mass training in the use of evidence-based clinical assessments, which can be as simple as standardized brief self-report instruments like the PHQ-9 [40] and PCL-5 [41].

Interventions should follow the principle of precision psychiatry, targeting the individual needs of those most affected, considering factors such as the type, duration, and intensity of trauma exposure, diagnosis, gender, and minority status. This targeted approach is crucial to mitigate the severe and prolonged impact on mental well-being in conflict settings.

Limited personnel limits access to mental health care. Task-shifting and task-sharing are strategies to improve access to evidence-based mental health care in resource-limited settings. Task-shifting involves transferring tasks from highly trained professionals (like clinical psychologists) to less specialized workers (community health workers or primary care providers). Task-sharing describes an approach wherein multiple providers share mental health care responsibilities. Task-shifting can help address mental health disparities and yield a strong return on investment [42]. Studies show that well-trained, supervised non-specialists can deliver effective short-term interventions for trauma, depression, PTSD, and other mental health issues [43–45]. Task-shifting models with adequate training and supervision have been found to improve mental health outcomes despite shortage of professionals. Although this approach can promote early symptom identification and access to care [46], it requires regulatory flexibility and a shift in viewpoint on integrated care [47]. With Ministry of Health support, Israel has begun training social workers and psychology college graduates to implement such models under the supervision of mental health specialists.

Some consequences of October 7 are novel and highly specific. For example, long traumatic confinement of hostages in underground tunnels will likely require ongoing care to overcome horrific effects of captivity, hunger, physical, sexual, and emotional abuse. Nonetheless, treatment research has long addressed generic common trauma-related disorders such as PTSD, depression, and anxiety, alone or comorbid, enabling developing and testing efficacious treatments. Meta-analyses [48–53] provide ample information on matching effective treatments to such syndromes. Large-scale training of cadres of Israeli clinicians, focusing on standardized assessments, personalized proven treatments, and effective monitoring, will provide access, expertise, and the ability to assess and effectively treat large number of trauma-exposed populations in acute need of highly focused, time-limited treatments.

## Conclusion

The scale of trauma and psychological distress following the October 7 attacks necessitates an urgent, systematic overhaul of Israeli mental health care. The burdens of PTSD, depression, anxiety, and related conditions will not abate without a concerted national effort to expand careful diagnosis and evidence-based treatment accessibility. Investing in clinician and task-shifting training, standardized assessments, targeted interventions and routine monitoring of their outcome is critical to mitigating long-term psychiatric consequences and fostering resilience. Without immediate action, the mental health crisis will deepen, further straining individuals, families, and the broader Israeli society.

## Data Availability

No datasets were generated or analysed during the current study.

## Abbreviations

PTSD: Posttraumatic Stress Disorder
PHQ-9: Patient Health Questionnaire-9
PCL-5: PTSD Checklist for DSM-5
